# Challenges and Considerations on Risk-Reducing Surgery in *BRCA1/2* Patients with Advanced Breast Cancer

**DOI:** 10.3390/curroncol28010050

**Published:** 2021-01-14

**Authors:** Leonor Vasconcelos de Matos, Leonor Fernandes, Pedro Louro, Ana Plácido, Manuel Barros, Fátima Vaz

**Affiliations:** 1 Medical Oncology Department, Hospital São Francisco Xavier, Centro Hospitalar Lisboa Ocidental, 1449-005 Lisboa, Portugal; lmrfernandes@chlo.min-saude.pt (L.F.); applacido@chlo.min-saude.pt (A.P.); 2 Familial Cancer Risk Clinic, Instituto Português de Oncologia de Lisboa Francisco Gentil, 1099-023 Lisboa, Portugal; pjplouro@gmail.com (P.L.); fvaz@ipolisboa.min-saude.pt (F.V.); 3 Faculty of Health Sciences, Universidade da Beira Interior, 6200-506 Covilhã, Portugal; 4 Oncology Unit, Centro Hospitalar do Oeste, 2500-176 Caldas da Rainha, Portugal; manuelbarros@choeste.min-saude.pt; 5 Medical Oncology Department, Instituto Português de Oncologia de Lisboa Francisco Gentil, 1099-023 Lisboa, Portugal

**Keywords:** breast cancer, ovarian cancer, hereditary cancer, BRCA, risk-reducing surgery

## Abstract

Cancer survivors harboring inherited pathogenic variants in the breast cancer (BC) susceptibility genes *BRCA1* or *BRCA2* are at increased risk of ovarian cancer (OC) and also of contralateral BC. For these women, risk-reducing surgery (RRS) may contribute to risk management. However, women with locally advanced or metastatic breast cancer (ABC) were excluded from clinical trials evaluating the benefit of these procedures in the *BRCA1/2* carriers, and thus, current guidelines do not recommend RRS in this specific setting. Although ABC remains an incurable disease, recent advances in treatment have led to increased survival, which, together with improvement in RRS techniques, raise questions about the potential role of RRS in the management of *BRCA1/2* ABC patients. When should RRS be discussed as an option for *BRCA1/2* patients diagnosed with ABC? To address this issue, we report two clinical cases that reflect new challenges in routine oncology practice. Team experience and patient motivations may shape multidisciplinary decisions in the absence of evidence-based data. A wise rationale may be the analysis of the competing risks of death by a previous ABC against risk of death by a secondary BC or OC, tailored to patient preferences.

## 1. Introduction

Breast cancer (BC) is the most frequently diagnosed cancer worldwide and the leading cause of cancer deaths in women [1]. Although most breast cancers are sporadic, inherited pathogenic variants in the breast cancer susceptibility genes *BRCA1* or *BRCA2* account for around 5% to 10% of BC diagnoses [2]. Carriers of pathogenic variants in these genes have an increased susceptibility to early-onset BC and ovarian cancer (OC) [3]. A substantial risk of contralateral disease [3] has been also described for unilateral *BRCA1/2* BC survivors. 

Risk-reducing surgery (RRS) is one of the procedures that may contribute to risk management in *BRCA1/2* women. Bilateral mastectomy (BM) reduces BC incidence by more than 90% and bilateral salpingo-oophorectomy (BSO), besides reducing OC incidence, leads to overall survival (OS) improvement [4,5]. BSO is recommended by age 35 to 40 or after completing childbearing [6], but in spite of its association with OS, women should be counselled regarding premature menopause and the potential for increased risks for bone and heart disease [7]. 

Most published studies concerning RRS in *BRCA* carriers include unaffected women or women with early BC [7,8,9]. Women with locally advanced or metastatic breast cancer (ABC) were excluded from trials evaluating the benefit of these procedures in the *BRCA1/2* setting and thus current recommendations do not approach RRS in such patients [6,8]. This is reasonable since ABC, although treatable, remains virtually an incurable disease, with an estimated 5-year survival of 27.4% [1], and risk of ABC-related death will most probably outweigh the risk of death by a second BC or by OC. 

Recent advances in ABC treatment are improving survival in these patients [10]. This change in the paradigm of BC prognosis, together with advances in RRS procedures, such as nipple-sparing mastectomy and laparoscopic BSO, can raise the question of the potential role of RRS in the setting of some ABC *BRCA1/2* patients. For highly selected women with a known *BRCA* mutation and ABC, can RRS be considered when disease control has been achieved and patients remain with no evidence of disease, sometimes even exceeding the progression-free survival (PFS) reported in clinical trials?

To address this issue, we report two clinical cases, which, due to advances in ABC management, are increasingly more common and pose new challenges in routine oncology clinical practice. 

## 2. Cases Description

### 2.1. Clinical Case 1

A 33-year-old woman, whose mother had died at 42 years of age from OC, was diagnosed in March 2007 with an invasive triple-negative carcinoma (28 mm) in the upper inner quadrant of the left breast. Complete staging showed a T2N + M1, with extensive metastatic liver disease. She started first-line sequential chemotherapy with 3 cycles of intravenous fluorouracil 500 mg/m^2^, epirubicin 75 mg/m^2^, and cyclophosphamide 500 mg/m^2^ (FEC-75), followed by 12 cycles of weekly paclitaxel 80 mg/m^2^. A sustained complete response (CR) was observed. This patient was identified with a *BRCA1* pathogenic variant (LRG_292t1:c.(4675+1_4676-1)_(5074+1_5475-1)del) and was kept under surveillance, and no surgery of the primary tumor nor RRS was performed. Six years and 8 months after the primary cancer diagnosis, she was diagnosed (February 2015) with another localized focus of triple-negative carcinoma in the left breast, cT3cN0M0. After neoadjuvant chemotherapy, since the patient was not motivated to have mastectomy, a left lumpectomy, followed by adjuvant radiotherapy, was performed. In February 2019, without any evidence of relapse, a risk-reducing BSO was performed. After almost 13 years (153 months) of her first ABC diagnosis, the patient remains with no evidence of disease.

### 2.2. Clinical Case 2

A 27-year-old woman, with no relevant personal or familial history, was diagnosed in March 2018 with a grade 3 invasive inflammatory carcinoma of the left breast, with a luminal B, epidermal growth factor receptor 2 (HER2) overexpression phenotype. Staging was performed with breast magnetic resonance imaging (MRI), body computed tomography (CT), bone scan, and fludeoxyglucose positron emission tomography (PET-FDG) and was classified as cT4dcN + cM1 (lymph node, bone, and liver metastasis). First-line treatment with intravenous docetaxel 75 mg/m^2^, trastuzumab 8 mg/kg loading dose followed by 6 mg/kg, and pertuzumab 840 mg loading dose followed by 420 mg, every 21 days, and bone-targeted therapy with monthly denosumab 120 mg subcutaneously, was started in May 2018. She was also referred to the reproduction services as well as to genetic counseling. After consenting for *BRCA1/2* testing, a pathogenic variant was diagnosed in the *BRCA2* gene (LRG_293t1:c.632-3C > G). After 6 cycles of therapy, in August 2018, a CR was observed in PET/CT. After 6 months of ovarian function suppression (OFS) with luteinizing hormone-releasing hormone (LH/RH) agonist and hormone therapy with the aromatase inhibitor exemestane, BSO was performed to maintain the stage IV hormone-receptor-positive BC patient under OFS. In September 2020, the patient remained under dual HER2 blockade (having completed 33 cycles of maintenance) and exemestane, with no evidence of disease (PFS of 25 months), and was highly motivated to discuss bilateral mastectomy, despite reluctance from the surgical team, arguing that no evidence sustains BM in the setting of ABC and the risk of BC relapse still outweighs the risk of a second BC.

## 3. Discussion

In reviewing these two cases we propose, as a rationale to approach the role of RRS in ABC, the analysis of the competing risks of death by a previous ABC against risk of death by a secondary BC or OC. This analysis should be tailored to the individual characteristics of each patient (with a previous complete remission and duration of follow-up being of the utmost relevance), family phenotype, and molecular diagnosis. 

*BRCA1* patients have a cumulative risk of BC by 80 years of age of around 72% and of OC of around 44%, while *BRCA2* patients have a cumulative risk of BC of around 69% and of OC of around 17% [11]. The risk of contralateral BC ranges between 60% and 80% [3] in these patients.

Women diagnosed with ABC, even after remission, are considered to have incurable disease. However, OS has improved over the last decade, particularly in some settings such as de novo disease, with a reported 3-year excess survival among these patients compared with that in studies prior to 1996 [12]. Some ABC women can live several years and multimodal approaches, including locoregional treatments with curative intent, can be considered for selected patients even if no specific prospective clinical trials have, so far, addressed this issue [13]. Patients with HER2 disease have seen the biggest change in median OS in the last years. The end-of-study analysis of the CLEOPATRA trial reported a median PFS of 20 months in the dual HER2 blockade arm, with an OS of 57.1 months, representing unprecedented results in the ABC setting [14]. Currently, OS of the overall ABC population is around 37 months [10]. 

These results outline the importance of an individualized approach to *BRCA1/2* ABC patients that are motivated for RRS. While patients’ preference should always be held into account, the potential benefits and pitfalls of RRS should also be explained to them. Besides survival and competing risks, discussion with the patients should also include the possible complications of RRS and of genetic testing, including adverse psychological outcomes brought by the recognition of risk for further cancers, besides recurrence.

Regarding case 1, the CR achieved with chemotherapy is not surprising, since a greater efficacy with higher CR rates in patients with a *BRCA1/2* BC has been previously described, owing to *BRCA1/2* functions in DNA repair and apoptosis [15,16,17]. Remarkable in this case, however, was the observation of a sustained long-term CR in the setting of a triple-negative ABC, a disease with an expected poor prognosis. When a second ipsilateral BC was diagnosed after a long disease-free interval from metastatic cancer, the possibility of BM was discussed with the patient. This procedure combines treatment and risk reduction surgery. In this discussion, not only the potential clinical benefits of BM, as sparing the patient the distress associated with a new possible BC diagnosis and the need of exposure to systemic treatments and resulting toxicities, but also the patient’s motivations regarding breast RRS should be borne in mind. The patient was not clearly motivated, and in the absence of guidelines or evidence of benefit in this particular context, conservative surgery was performed. It should also be considered for the discussion that although there is evidence of a survival impact of BSO in *BRCA1/2* patients, conflicting evidence exists regarding RR mastectomy [18]. In the case presented, after two more years without cancer relapse, the patient consented for BSO, which is also in accordance with previous data that report a higher adherence of *BRCA1/2* patients to BSO than to RR mastectomy [19].

Concerning case 2, this young woman is highly motivated for bilateral mastectomy, but her follow-up is still insufficient to propose the procedure. Despite her PFS having already surpassed the median PFS reported in the CLEOPATRA trial [14], a longer follow-up may be needed to manage expectations regarding the delicate balance between risk of relapse and risk of a new cancer diagnosis. In this case, BSO was performed, not as an RRS but with the rationale of OFS for a premenopausal woman, as recommended in current guidelines [13]. Surgery has shown no difference in outcomes compared with chemical OFS and with a slightly better tolerance and toxicity profile [18]. Regarding RR surgery in ABC, neither is discussed in current guidelines or has been previously described in the literature. Patient expectations in this case were addressed by negotiating a longer follow-up before deciding on bilateral mastectomy, since the risk of relapse was still considered to predominate over the risk of a second cancer diagnosis.

While discussing two specific clinical cases to support our proposal to analyze competing risks regarding RR surgeries in the setting of ABC, we understand that specific clinicopathological characteristics had an impact on the patients’ outcomes. While the first case may represent a large subgroup of *BRCA1* BC, due to its triple-negative phenotype [19], the majority of *BRCA1/2* BC lack HER2 overexpression. However, the reported rates of HER2 overexpression are higher for *BRCA2* than for *BRCA1* BC, ranging between 3% and 17% [19,20,21]. For both BC molecular profiles, the emergence of new treatment options has led to promising survival results, which adds relevance to the discussion brought by these brief reports. In triple-negative ABC, the combination of immune checkpoint inhibitors and chemotherapy may lead to a 10-month improvement in OS, and poly (ADP-ribose) polymerase (PARP) inhibitors in the setting of germline *BRCA* mutation have also shown PFS improvements [22,23,24]. In the setting of HER2 BC, the treatment landscape has also been noticeably changed, not only with double blockade in epidermal growth factor receptor 2 (HER2)-positive ABC, which has led to better OS in these patients [10], but also with the advent of novel agents that have recently emerged, stretching survival expectations even further [25,26,27,28,29].

## 4. Conclusions

To the best of our knowledge, no reports in the literature exist regarding the percentage of BRCA1/2 ABC patients that could be considered for RRS. However, challenges brought by cases similar to these will be increasingly more frequent in BC clinics, since genetic testing is now recommended in a larger number of BC patients [30]. Patient motivations about RRS after long complete remissions may shape multidisciplinary and individual tailored decisions in the absence of evidence-based data. With the survival impact of therapeutic advances, we owe our ABC survivors the duty to address their needs. We propose that RRS may be discussed for selected ABC BRCA1/2 patients after complete and sustained response of their ABC to systemic or multimodality treatment. At the present time, competing risks (of relapse versus a new primary cancer) should be carefully analyzed for each patient. Hopefully, more research on this subject will bring new evidence to support best patient-centered care decisions.
